# Enhancing 3D semantic scene completion with refinement module

**DOI:** 10.3389/fnbot.2026.1768219

**Published:** 2026-03-06

**Authors:** Dunxing Zhang, Jiachen Lu, Han Yang, Lei Bao, Bo Song

**Affiliations:** 1 Chair of Robotics, Artificial Intelligence and Real-time Systems, Technical University of Munich, Munich, Germany; 2 National Science Center for Earthquake Engineering, Tianjin University, Tianjin, China; 3 School of Civil Engineering, Tianjin University, Tianjin, China

**Keywords:** plug-and-play, PNAM, refinement, semantic scene completion, vison-language guidance

## Abstract

We propose ESSC-RM, a plug-and-play Enhancing framework for Semantic Scene Completion with a Refinement Module, which can be seamlessly integrated into existing semantic scene completion (SSC) models. ESSC-RM operates in two phases: a baseline SSC network first produces a coarse voxel prediction, which is subsequently refined by a 3D U-Net–based Prediction Noise-Aware Module (PNAM) and Voxel-level Local Geometry Module (VLGM) under multiscale supervision. Experiments on SemanticKITTI show that ESSC-RM consistently improves semantic prediction performance. When integrated into CGFormer and MonoScene, the mean IoU increases from 16.87 to 17.27% and from 11.08 to 11.51%, respectively. These results demonstrate that ESSC-RM serves as a general refinement framework applicable to a wide range of SSC models. Project page: https://github.com/LuckyMax0722/ESSC-RM and https://github.com/LuckyMax0722/VLGSSC.

## 1 Introduction

Accurate 3D scene understanding is fundamental to autonomous driving, robotics, and embodied perception, where downstream tasks such as detection, reconstruction, mapping, and planning rely on complete geometric and semantic representations of the environment (Guo et al., 2019; Yurtsever et al., 2020; Cao et al., 2022; Ma et al., 2022; Zhao H. et al., 2024; Cao et al., 2024a). However, real-world sensors (LiDAR and RGB cameras) provide only sparse, noisy, and partial observations due to occlusions, limited resolution, restricted field of view, and missing depth information, resulting in incomplete voxelized scenes (Roldão et al., 2021; Cao et al., 2024b). To address this, 3D semantic scene completion (SSC) aims to jointly infer voxel occupancy and semantic labels, a task first formalized by SSCNet (Song et al., 2016).

Despite extensive progress in both LiDAR-based (Roldão et al., 2020; Yan et al., 2020; Xia et al., 2023; Jang et al., 2024) and vision-based SSC (Cao and de Charette, 2021; Li Y. et al., 2023; Jiang et al., 2024; Tang et al., 2023), a considerable gap remains between predictions and ground truth. LiDAR-based models suffer from sparsity; BEV-based methods (Yang et al., 2021) lose fine-grained details; RGB-based approaches degrade due to depth ambiguity and unclear 2D–3D projection (Lee et al., 2024); and distillation pipelines depend heavily on task-specific teacher designs (Xia et al., 2023). Moreover, SSC architectures differ substantially, making it difficult to develop a unified refinement strategy that generalizes across models without modifying their internal structures.

To bridge these limitations, this paper proposes ESSC-RM, a unified coarse-to-fine refinement framework that directly enhances the voxel predictions of arbitrary SSC models. ESSC-RM performs multi-scale geometric–semantic aggregation, integrates auxiliary priors, and introduces a model-agnostic refinement pipeline that requires no architectural modification to the baseline. It supports both end-to-end joint training and fully independent plug-and-play deployment.

The main contributions of this paper are as follows:

- We introduce ESSC-RM, a general refinement framework designed to improve heterogeneous SSC baselines via coarse-to-fine multi-scale error reduction, applicable to both LiDAR-based and vision-based methods.
- We develop two complementary training paradigms: a *joint training* mode that co-optimizes the refinement and baseline networks, and a *separate training* mode enabling true plug-and-play enhancement without modifying the original SSC architecture.
- We propose a neighborhood-attention-based multi-scale aggregation module that adaptively fuses geometric and semantic features, improving voxel-level reasoning across scales.
- We introduce a novel vision–language guidance module that injects text-derived semantic priors to compensate for missing geometric cues and ambiguous visual projections, enhancing cross-modal scene understanding.
- Extensive experiments on SemanticKITTI (Behley et al., 2019) demonstrate that ESSC-RM consistently improves strong baselines such as CGFormer and MonoScene, validating its generality, flexibility, and effectiveness.

## 2 Related work

In this section, we review *LiDAR- and camera-based 3D perception*, then summarize advances in *3D SSC*, and finally discuss recent progress in *vision–language models (VLMs)* and *text-driven multimodal fusion*.

### 2.1 LiDAR-based 3D perception

LiDAR provides accurate 3D geometry for autonomous driving perception, enabling detection, tracking, and mapping, and has become a core sensing modality (Guo et al., 2019; Yurtsever et al., 2020; Ma et al., 2022; Zhao H. et al., 2024; Wu et al., 2022; Lin and Wu, 2025).

Early point-based and voxel-based detectors—PointNet (Qi et al., 2016), VoxelNet (Zhou and Tuzel, 2017), SECOND (Yan et al., 2018), PointPillars (Lang et al., 2018), PointRCNN (Shi et al., 2018), PV-RCNN (Shi et al., 2019), and Voxel R-CNN (Deng et al., 2020)—established effective feature extraction paradigms. Tracking frameworks such as AB3DMOT (Weng et al., 2020; Cho and Kim, 2023) leverage motion models and geometric association. Semantic segmentation approaches including PointNet++ (Qi et al., 2017), RangeNet++ (Milioto et al., 2019), and Cylinder3D (Zhou et al., 2020) demonstrate point-based, projection-based, and cylindrical-voxel inference strategies.

### 2.2 Camera-based 3D perception

Camera-based perception offers a cost-efficient alternative with rich semantic cues. Monocular approaches extend 2D detectors (Brazil and Liu, 2019; Duan et al., 2019; Manhardt et al., 2018) or rely on pseudo-depth and geometric priors (Xu and Chen, 2018; Wang et al., 2018; Zia et al., 2014; Mousavian et al., 2016; Hu et al., 2018), yet remain affected by depth ambiguity. Stereo-based methods (Chang and Chen, 2018; Li et al., 2019; You et al., 2019; Chen et al., 2020) mitigate this by enforcing geometric consistency (Mao et al., 2023).

With multi-camera setups becoming standard, multi-view 3D detection methods have evolved rapidly. LSS-based pipelines (Philion and Fidler, 2020; Huang et al., 2021) lift image features to Bird's-Eye View (BEV), while transformer-based designs such as DETR3D (Wang et al., 2021) and BEVFormer (Li Z. et al., 2022) aggregate cross-view features using 3D object queries. Spatiotemporal attention mechanisms (Vaswani et al., 2017; Doll et al., 2022; Mao et al., 2023) further enhance robustness.

### 2.3 Semantic scene completion

SSC jointly predicts occupancy and voxel-level semantics. SSCNet (Song et al., 2016) established the task on indoor data (Silberman et al., 2012); outdoor datasets such as KITTI and SemanticKITTI (Geiger et al., 2012; Behley et al., 2019, 2021; Li et al., 2024) introduce sparsity and large-scale variability.

### 2.4 Vision–language models

Vision–language models (VLMs) provide strong semantic priors through aligned image–text representations (Liu et al., 2025). CLIP (Radford et al., 2021) and EVACLIP (Sun et al., 2023a,b) learn powerful contrastive embeddings, while LongCLIP (Zhang et al., 2024) and JinaCLIP (Xiao et al., 2024a; Koukounas et al., 2024) improve long-text modeling.

Models such as BLIP2 (Li J. et al., 2023), InstructBLIP (Dai et al., 2023), MiniGPT-4 (Zhu et al., 2024), and LLaVA (Liu H. et al., 2023; Liu et al., 2024) leverage frozen Large Language Models (LLMs) to build efficient multimodal reasoning pipelines (OpenAI et al., 2024). Text-conditioned segmentation models such as LSeg (Li B. et al., 2022) and Grounded-SAM (Ren et al., 2024) further highlight the utility of text in perception tasks (Liu S. et al., 2023; Kirillov et al., 2023).

### 2.5 Multimodal fusion and text modality

Multimodal fusion traditionally combines 3D geometry (LiDAR, stereo) with rich 2D semantics. With the emergence of LLMs and VLMs, text has become a scalable, low-cost semantic modality for describing road scenes (Li and Tang, 2024; Liu et al., 2025).

Attention-based fusion (Vaswani et al., 2017; Cao et al., 2021)—as in (Xu et al. 2020), (Cao et al. 2024c), and (Wang et al. 2026)—captures long-range cross-modal dependencies but can be computationally heavy. Learnable fusion strategies such as Text-IF (Yi et al., 2024) and VLScene (Wang et al., 2025) use trainable coefficients to balance visual and linguistic cues.

## 3 Methodology

**Figure 1:**
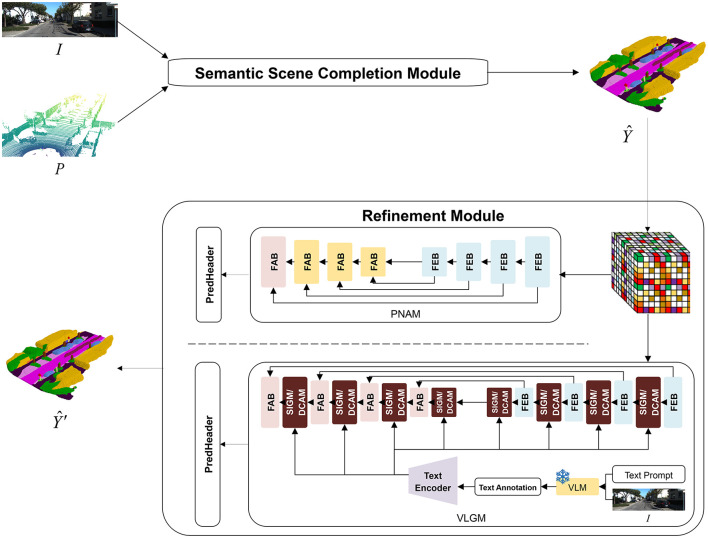
Overall architecture of ESSC-RM. An SSC backbone first predicts a coarse semantic voxel grid from image and/or LiDAR input. The refinement module embeds the discrete voxel labels into continuous features and processes them with a 3D U-Net enhanced by PNAM and VLGM, finally producing a refined semantic volume ${\hat{\text{Y}}}^{\prime }$.

ESSC-RM refines the coarse voxel predictions produced by any SSC backbone. We now present the problem formulation and describe the architecture components of our refinement module, including the 3D U-Net backbone, the progressive neighborhood attention module (PNAM), and the vision–language guidance module (VLGM), as illustrated in Figure 1.

### 3.1 Problem statement

Given an RGB image ${\text{I}}_{t}\in {\mathbb{R}}^{H\times W\times 3}$ and a LiDAR point cloud ${\text{P}}_{t}\in {\mathbb{R}}^{N\times 3}$ at time *t*, 3D SSC aims to predict a dense semantic voxel grid ${\hat{\text{Y}}}_{t}\in {\left\{c_{0},c_{1},\ldots ,c_{C}\right\}}^{H\times W\times Z}$ defined in the vehicle coordinate system, where each voxel is either empty (*c*_0_) or belongs to one of the *C* semantic classes {*c*_1_, …, *c*_*C*_} and *H, W, Z* denote the voxel grid dimensions. A standard SSC backbone learns ${\hat{\text{Y}}}_{t}=f_{\theta }\left({\text{I}}_{t},{\text{P}}_{t}\right)$, but the coarse prediction ${\hat{\text{Y}}}_{t}$ often exhibits broken surfaces, incomplete structures, and semantic confusions. We therefore introduce a refinement module *g*_ϕ_ that treats ${\hat{\text{Y}}}_{t}$ as a noisy discrete volume and outputs a refined prediction ${\hat{\text{Y}}}_{t}^{\prime }=g_{\phi }\left({\hat{\text{Y}}}_{t},\text{aux}\right)$, where *aux* denotes additional cues (multi-scale voxel features and text semantics) extracted within the refinement module. The objective is to bring ${\hat{\text{Y}}}_{t}^{\prime }$ closer to the ground truth **Y**_*t*_ in both geometry and semantics while remaining compatible with heterogeneous SSC backbones.

### 3.2 Overall architecture

As shown in Figure 1, ESSC-RM has two decoupled parts:

- SSC backbone: maps (**I**_*t*_, **P**_*t*_) to a coarse voxel grid ${\hat{\text{Y}}}_{t}$.
- Refinement module: operates purely in voxel space, refining ${\hat{\text{Y}}}_{t}$ into ${\hat{\text{Y}}}_{t}^{\prime }$ using multi-scale U-Net features, neighborhood attention, and vision–language guidance.

This separation allows us to plug in backbones of different quality while focusing the design of *g*_ϕ_ on correcting geometric and semantic errors at the voxel level using additional structural and semantic cues.

### 3.3 SSC backbone

ESSC-RM is model-agnostic and can refine the output of any SSC backbone. To demonstrate generality, we instantiate two monocular SSC models with different coarse prediction qualities: CGFormer (Tang et al., 2023) and MonoScene (Cao and de Charette, 2021). CGFormer represents a strong backbone with accurate voxel lifting, while MonoScene produces notably noisier volumes, providing a more challenging setting for refinement. All architectural details follow the original papers, as our refinement module does not modify or depend on the internal design of the backbone.

### 3.4 3D U-Net refinement backbone

**Figure 2:**
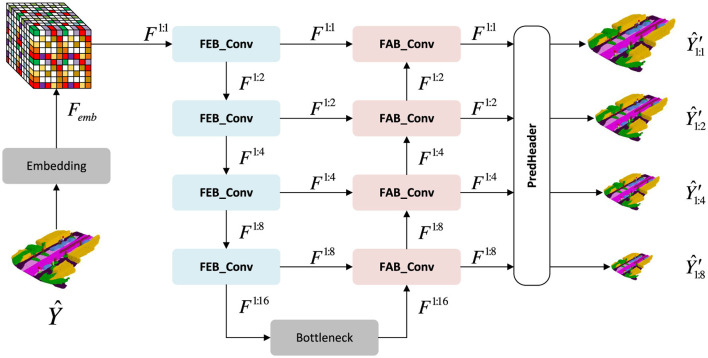
Three-dimensional U-shaped neural network (3D U-Net) backbone used in the refinement module. The encoder consists of stacked feature encoding blocks (FEBs) that progressively downsample the input and extract multi-scale features, a bottleneck aggregates global context, and the decoder consists of stacked feature aggregation blocks (FABs) that progressively upsample the features and predict semantic voxel outputs at four spatial scales.

**Figure 3:**
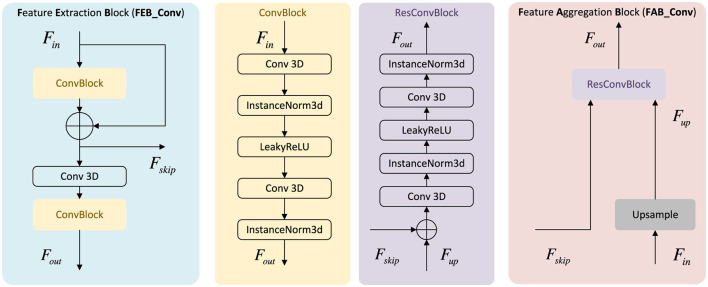
Core components of the three-dimensional U-shaped neural network (3D U-Net). The feature encoding block (FEB) applies three-dimensional convolutions, normalization, and residual connections to encode geometric and semantic information, while the feature aggregation block (FAB) upsamples low-resolution features and fuses them with skip connections to recover fine spatial details.

The refinement module receives the coarse discrete volume $\hat{\text{Y}}$ and must (i) map it into a continuous feature space; (ii) aggregate multi-scale contextual information; and (iii) reconstruct a refined voxel grid ${\hat{\text{Y}}}^{\prime }$. To accomplish these steps, we adopt a three-dimensional U-shaped neural network (3D U-Net) backbone (Çiçek et al., 2016; Ronneberger et al., 2015), whose overall encoder–bottleneck–decoder structure is illustrated in Figure 2. The specific computational blocks that constitute the encoder and decoder, namely the feature encoding block (FEB) and the feature aggregation block (FAB), are detailed in Figure 3.

#### 3.4.1 Voxel embedding and encoder–decoder

We first embed the discrete labels of $\hat{\text{Y}}$ into a continuous feature map:


$$\begin{array}{l} F_{\text{emb}}=\text{Embedding}\left(\hat{\text{Y}}\right), \end{array}$$ (1)

where $F_{\text{emb}}\in {\mathbb{R}}^{C\times H\times W\times Z}$. A 1 × 1 × 1 3D convolution then produces the input feature:


$$\begin{array}{l} F_{\text{in}}={\text{Conv}}_{\text{in}}\left(F_{\text{emb}}\right). \end{array}$$ (2)

The encoder uses four stacked feature encoding blocks (FEBs; Figure 3) to extract multi-scale features *F*^1:*s*^ at progressively lower resolutions. For a voxel grid of size *H*×*W*×*Z* and feature dimension *G*, the encoder outputs


$$\begin{array}{l} F^{1:s}\in {\mathbb{R}}^{G\times \frac{H}{s}\times \frac{W}{s}\times \frac{Z}{s}},\;s\in \left\{1,2,4,8,16\right\}. \end{array}$$ (3)

A bottleneck processes *F*^1:16^, and the decoder then upsamples via four stacked feature aggregation blocks (FABs), followed by a shared prediction head that produces voxel logits at multiple scales:


$$\begin{array}{l} {Ŷ}_{1:s}^{\prime }\in {\mathbb{R}}^{C\times \frac{H}{s}\times \frac{W}{s}\times \frac{Z}{s}},\;s\in \left\{1,2,4,8\right\}, \end{array}$$ (4)

where *C* is the number of semantic classes. At inference time, we use


$$\begin{array}{l} {\hat{\text{Y}}}^{\prime }=\arg\underset{c}{\max}{Ŷ}_{1:1,c}^{\prime } \end{array}$$ (5)

as the final refined semantic voxel prediction.

#### 3.4.2 Feature encoding block (FEB)

Each FEB refines features at a given scale and produces both a skip feature and a downsampled feature. As in Figure 3, an FEB applies two 3D convolutions with InstanceNorm3D (Ulyanov et al., 2016) and LeakyReLU (Xu et al., 2015), followed by a residual skip and a stride-2 convolution:


$$\{\begin{array}{l} F_{\text{skip}}^{1:\ell }=\text{ConvBlock}(F_{\text{in}}^{1:\ell })+F_{\text{in}}^{1:\ell },\\ F^{1:2\ell }={\text{Conv}}_{2\times 2\times 2}(F_{\text{skip}}^{1:\ell }),\\ F_{\text{out}}^{1:2\ell }=\text{ConvBlock}(F^{1:2\ell }), \end{array}\;\forall \ell \in \{1,2,4,8\}.$$ (6)

#### 3.4.3 Feature aggregation block (FAB) and multi-scale supervision

Each FAB upsamples low-resolution features and fuses them with encoder skip features:


$$\{\begin{array}{l} F_{\text{up}}^{1:2\ell }=\text{UpSample}(F_{\text{in}}^{1:2\ell }),\\ F_{\text{out}}^{1:\ell }=\text{ResConvBlock}(F_{\text{skip}}^{1:\ell },F_{\text{up}}^{1:2\ell }), \end{array}\;\forall \ell \in \{1,2,4,8\}.$$ (7)

Following PaSCo (Cao A.-Q. et al., 2024), each decoder feature map $F_{\text{out}}^{1:\ell }$ is mapped to logits by a 1 × 1 × 1 3D convolution:


$$\begin{array}{l} {Ŷ}_{1:\ell }^{\prime }=\text{PredHead}\left(F_{\text{out}}^{1:\ell }\right),\;\forall \ell \in \left\{1,2,4,8\right\}, \end{array}$$ (8)

and all scales are supervised during training. This encourages coarse-to-fine refinement and stabilizes optimization.

### 3.5 Progressive neighborhood attention module (PNAM)

Purely convolutional decoders aggregate context only within fixed local windows, limiting their ability to capture long-range and structure-aware voxel relations. To address this, we integrate the Progressive Neighborhood Attention Module (PNAM) (Liu T. et al., 2023) into the decoder of our refinement network.

**Figure 4:**
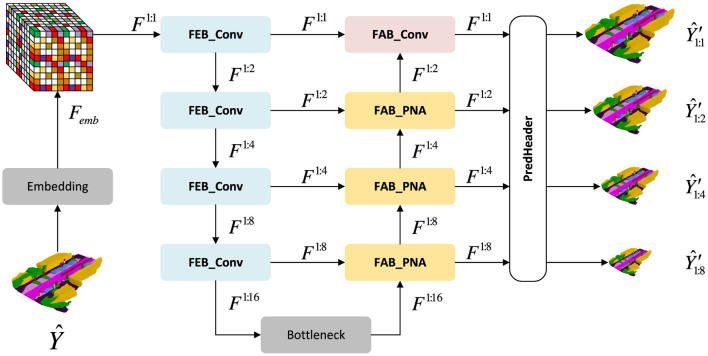
Progressive neighborhood attention U-Net (PNA U-Net) (Liu T. et al., 2023). PNAM replaces the feature aggregation blocks (FABs) at intermediate scales (1:2, 1:4, 1:8) with attention-based FABs, while the finest-scale FAB remains convolutional for efficiency.

As illustrated in Figure 4, the FABs at scales 1:2, 1:4, and 1:8 are replaced with PNA-based FABs, while the finest-scale FAB remains convolutional for efficiency. PNAM enhances multi-scale voxel reasoning by combining global self-attention (Vaswani et al., 2017) with localized neighborhood aggregation (Hassani and Shi, 2022; Hassani et al., 2023, 2024).

#### 3.5.1 PNA-based feature aggregation block

**Figure 5:**
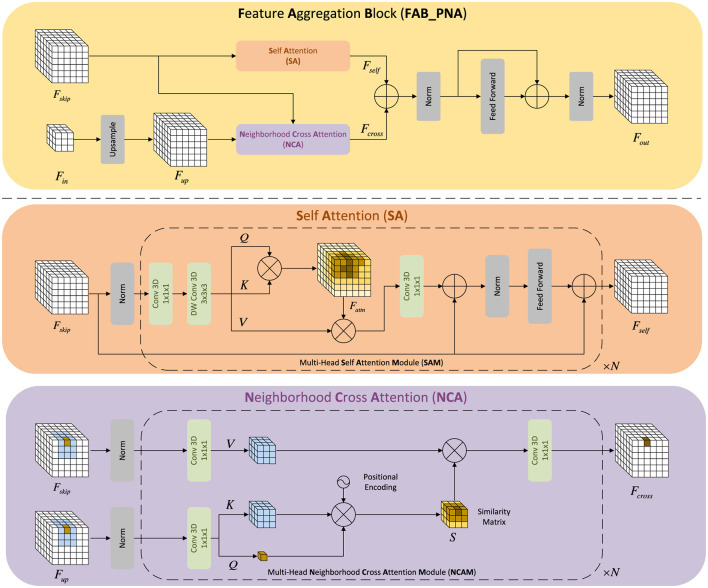
Core components of the PNA-based feature aggregation block (FAB). Each block combines self-attention (SA) and neighborhood cross-attention (NCA) to jointly model long-range dependencies and local geometric consistency.

As illustrated in Figure 5, a PNA-based FAB consists of two branches: (1) a self-attention (SA) branch operating on *F*_up_; and (2) a neighborhood cross-attention (NCA) branch operating between *F*_skip_ and *F*_up_.

Given the upsampled feature $F_{\text{up}}^{1:\ell }$ and the corresponding skip feature $F_{\text{skip}}^{1:\ell }$, the two attention responses are computed as:


$$F_{\text{self}}^{1:\ell }=\text{SA}\left(F_{\text{up}}^{1:\ell }\right),\;F_{\text{cross}}^{1:\ell }=\text{NCA}\left(F_{\text{skip}}^{1:\ell },F_{\text{up}}^{1:\ell }\right),$$

for ℓ ∈ {2, 4, 8}. The outputs are fused and refined via normalization and a lightweight feed-forward network (FFN):


$$F_{\text{out}}^{1:\ell }=\text{FFN}(\text{Norm}\left(F_{\text{self}}^{1:\ell }+F_{\text{cross}}^{1:\ell }\right)).$$

#### 3.5.2 Self-attention (SA)

SA refines the upsampled voxel features by capturing long-range dependencies. Following the standard multi-head attention formulation (Vaswani et al., 2017), we use 1^3^ and depthwise 3^3^ convolutions to compute *Q, K, V*, followed by attention and a residual FFN. This propagates global geometric–semantic cues, compensating for missing structures in the coarse prediction.

#### 3.5.3 Neighborhood cross-attention (NCA)

NCA enforces local geometric consistency. Inspired by the NATTEN family of neighborhood attention operators (Hassani and Shi, 2022; Hassani et al., 2023, 2024), it restricts attention to a 3D neighborhood window, enabling each voxel to aggregate high-confidence structural cues from spatially adjacent voxels. This makes PNAM particularly effective at restoring fine structures such as object boundaries and thin geometry.

Overall, PNAM strengthens the refinement network's ability to jointly model global context and local voxel continuity across scales.

### 3.6 Vision–language guidance module (VLGM)

**Figure 6:**
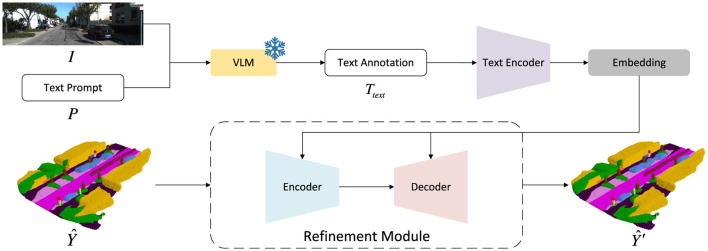
VLGM pipeline. A frozen VLM produces a free-form scene description, which is encoded and fused with voxel features.

Even with stronger voxel–voxel reasoning, SSC remains ambiguous in occluded or sparsely observed regions. To inject high-level scene priors—such as road layout, object co-occurrence patterns, or typical urban structures—we introduce the Vision–Language Guidance Module (VLGM). As illustrated in Figure 6, the module leverages a frozen vision–language model (VLM) to produce a free-form scene description, whose textual semantics are encoded and fused into the voxel refinement pipeline.

#### 3.6.1 Text acquisition and semantic encoding

Given an input image *I* and prompt *P*, a frozen VLM such as LLaVA (Liu H. et al., 2023; Liu et al., 2024) or InstructBLIP (Dai et al., 2023) generates a scene description


$$T_{\text{text}}=\text{VLM}\left(I,P\right),$$

which is precomputed offline to avoid training overhead.

To capture different levels of textual semantics, we employ two complementary encoders. (1) JinaCLIP (Xiao et al., 2024a; Koukounas et al., 2024) extracts a global embedding


$$F_{\text{text}}^{\text{JinaCLIP}}=\text{JinaCLIP}\left(T_{\text{text}}\right),$$

providing holistic scene cues; and (2) A Q-Former (Li J. et al., 2023) produces token-level embeddings


$$F_{\text{text}}^{\text{Q-Former}}=\text{Q-Former}\left(T_{\text{text}}\right),$$

which enable fine-grained cross-modal alignment. This design follows instruction-style prompting practices used in (Dai et al. 2023); (Liu et al. 2025).

#### 3.6.2 Text–voxel fusion modules

To integrate text cues into voxel refinement, we build a Text U-Net by inserting lightweight fusion blocks after each FEB and FAB. Each fusion block consists of two components:

##### 3.6.2.1 Semantic interaction guidance module (SIGM)

Following Text-IF (Yi et al., 2024), global JinaCLIP features are mapped to affine parameters (γ_*m*_, β_*m*_) via MLPs. Voxel features are modulated as


$$F_{\text{out}}=\left(1+\gamma_{m}\right)⊙F_{\text{in}}+\beta_{m},$$

injecting scene-level priors that guide early geometric reasoning.

##### 3.6.2.2 Dual cross-attention module (DCAM)

Inspired by BLIP-2 (Li J. et al., 2023), SAM (Kirillov et al., 2023), and MultiRAtt-RSSC (Cai et al., 2024), DCAM alternates self- and cross-attention between Q-Former tokens and voxel features. Text self-attention yields $F_{\text{text}}^{\text{attn}}$, followed by text-to-voxel cross-attention producing $F_{\text{text}}^{\text{enhanced}}$, and voxel-to-text cross-attention generating $F_{\text{voxel}}^{\text{enhanced}}$. A residual update produces


$$F_{\text{out}}=\text{LayerNorm}\left(F_{\text{voxel}}^{\text{enhanced}}+F_{\text{in}}\right).$$

SIGM injects global scene priors (e.g., “urban street with parked vehicles”), while DCAM provides fine-grained token-level alignment. As visualized in Figure 6, the two components operate synergistically to improve geometric completeness and semantic coherence, especially in occluded and ambiguous regions.

### 3.7 Loss function

ESSC-RM performs coarse-to-fine refinement across multiple spatial scales. We therefore supervise both voxel-wise predictions and scene-level consistency using two complementary terms: a class-weighted cross-entropy loss and the scene–class affinity loss (SCAL) (Cao and de Charette, 2021; Tang et al., 2023). This combination stabilizes multi-scale refinement while encouraging globally coherent semantics.

#### 3.7.1 Cross-entropy loss

At each refinement scale *l*, voxel predictions are supervised using a class-weighted cross-entropy:


$$\begin{array}{l} L_{l}=-\frac{1}{C}\overset{N}{\underset{i=0}{\sum }}\overset{C}{\underset{c=0}{\sum }}w_{c}y_{i,c}\log\frac{\exp\left({ŷ}_{i,c}^{\prime }\right)}{\sum_{c^{\prime }=0}^{C}\exp\left({ŷ}_{i,c^{\prime }}^{\prime }\right)}, \end{array}$$ (9)

where ŷ′ denotes refinement logits and *w*_*c*_ compensates for class imbalance (Roldão et al., 2020). Aggregating all scales yields:


$$\begin{array}{l} L_{ce}=\overset{L}{\underset{l=0}{\sum }}L_{l}. \end{array}$$ (10)

#### 3.7.2 Scene–class affinity loss (SCAL)

To promote globally consistent refinement—particularly under sparsity or ambiguous projections—we adopt SCAL (Cao and de Charette, 2021), which optimizes class-wise precision (*P*_*c*_), recall (*R*_*c*_), and specificity (*S*_*c*_). Let *p*_*i*_ denote the ground-truth class for voxel *i*, and ${\hat{p}}_{i,c}$ the predicted probability for class *c*. Using Iverson brackets ⟦·⟧, the metrics are:


$$\begin{array}{l} P_{c}\left(\hat{p},p\right)=\log\frac{\sum_{i}{\hat{p}}_{i,c}⟦p_{i}=c⟧}{\sum_{i}{\hat{p}}_{i,c}}, \end{array}$$ (11)

$$\begin{array}{l} R_{c}\left(\hat{p},p\right)=\log\frac{\sum_{i}{\hat{p}}_{i,c}⟦p_{i}=c⟧}{\sum_{i}⟦p_{i}=c⟧}, \end{array}$$ (12)

$$\begin{array}{l} S_{c}\left(\hat{p},p\right)=\log\frac{\sum_{i}\left(1-{\hat{p}}_{i,c}\right)\left(1-⟦p_{i}=c⟧\right)}{\sum_{i}\left(1-⟦p_{i}=c⟧\right)}. \end{array}$$ (13)

The per-scale affinity loss is:


$$\begin{array}{l} L_{l}\left(\hat{p},p\right)=-\frac{1}{C}\overset{C}{\underset{c=1}{\sum }}(P_{c}\left(\hat{p},p\right)+R_{c}\left(\hat{p},p\right)+S_{c}\left(\hat{p},p\right)). \end{array}$$ (14)

SCAL is applied to both semantic and geometric predictions across all refinement scales:


$$\begin{array}{l} L_{scal}^{sem}=\overset{L}{\underset{l=0}{\sum }}L_{l}\left({ŷ}^{\prime },y\right), \end{array}$$ (15)

$$\begin{array}{l} L_{scal}^{geo}=\overset{L}{\underset{l=0}{\sum }}L_{l}\left({ŷ}^{\prime ,geo},y^{geo}\right). \end{array}$$ (16)

#### 3.7.3 Overall objective

The total training loss is:


$$\begin{array}{l} L={\text{λ}}_{ce}L_{ce}+{\text{λ}}_{scal}^{geo}L_{scal}^{geo}+{\text{λ}}_{scal}^{sem}L_{scal}^{sem}, \end{array}$$ (17)

with all coefficients set to 1 in our experiments, providing a balanced supervision over voxel-wise accuracy, geometric completion, and scene-level semantic consistency.

## 4 Experiment

This section evaluates ESSC-RM on the SemanticKITTI benchmark (Behley et al., 2019, 2021). We first describe the experimental setup (datasets, metrics, and implementation), then report quantitative and qualitative results on strong and weak semantic scene completion baselines (CGFormer and MonoScene). Comprehensive ablation studies that analyze the refinement framework, the neighborhood-attention-based aggregation module, and the vision–language guidance module are provided in the Supplementary material.

### 4.1 Experimental setup

#### 4.1.1 Datasets

We adopt the SemanticKITTI semantic scene completion benchmark (Behley et al., 2019, 2021), which extends the KITTI odometry dataset (Geiger et al., 2012) with dense semantic labels for each LiDAR scan. The dataset contains 22 outdoor sequences; following the official split, sequences 00–07 and 09–10 are used for training, 08 for validation, and 11–21 as a hidden test set.

For semantic scene completion, a 3D volume around the ego-vehicle is considered: 51.2m in front, 25.6m to each side (total width 51.2m), and 6.4m in height (Behley et al., 2019). This volume is voxelized into a 256 × 256 × 32 grid with voxel size 0.2m^3^. Each voxel is assigned one of 20 classes (19 semantic classes and 1 free-space), obtained by voxelizing aggregated, registered semantic point clouds (Li Y. et al., 2023).

We conduct all experiments on SemanticKITTI, following its established voxelization protocol and official evaluation scripts, which provides a standardized testbed for semantic scene completion.

#### 4.1.2 Evaluation metrics

We follow standard practice (Cao and de Charette, 2021; Li Y. et al., 2023; Tang et al., 2023) and report intersection-over-union (IoU) for 3D scene completion (SC) and mean intersection-over-union (mIoU) for semantic scene completion (SSC).

For SC, evaluation is binary (occupied vs. free) and uses IoU over the occupancy grid:


$$\begin{array}{l} \text{IoU}=\frac{\text{completion\_TP}}{\text{completion\_TP}+\text{completion\_FP}+\text{completion\_FN}}, \end{array}$$ (18)

where TP, FP, and FN denote true positives, false positives, and false negatives on the occupancy grid.

For SSC, we evaluate per-class IoU over *C* = 19 semantic classes and report mean IoU:


$$\begin{array}{l} \text{mIoU}=\frac{1}{C}\overset{C}{\underset{c=1}{\sum }}\frac{{\text{TP}}_{c}}{{\text{TP}}_{c}+{\text{FP}}_{c}+{\text{FN}}_{c}+\epsilon }, \end{array}$$ (19)

where TP_*c*_, FP_*c*_, and FN_*c*_ are computed for class *c*, and evaluation is carried out in known space as in (Roldão et al., 2021). IoU primarily reflects geometric completion quality, whereas mIoU captures voxel-wise semantic accuracy; both are reported to assess overall scene understanding.

#### 4.1.3 Implementation details

We consider two training paradigms for ESSC-RM: (1) joint training, where the semantic scene completion backbone is switched to inference mode while the refinement module is trained on-the-fly from its predictions; and (2) separate training, where semantic scene completion predictions are pre-computed and stored, and the refinement module is trained purely as a plug-and-play post-processor without modifying the original semantic scene completion architecture.

Unless otherwise stated, experiments are conducted on two NVIDIA RTX A5000 GPUs, with 10 epochs and a batch size of 1 per GPU. We use AdamW (Loshchilov and Hutter, 2017) with β_1_ = 0.9, β_2_ = 0.99, and a peak learning rate of 5 × 10^−5^. A cosine schedule (Smith and Topin, 2017) with 5% warm-up is applied. The refinement module follows a 3D U-Net (Çiçek et al., 2016) backbone; encoder and decoder feature-enhancement blocks (FEB/FAB) are adapted from SemCity (Lee et al., 2024), and neighborhood-attention-based variants from NATTEN (Hassani and Shi, 2022; Hassani et al., 2023, 2024) and PNA (Liu T. et al., 2023). The vision–language guidance module (VLGM) uses frozen vision–language models [InstructBLIP (Li J. et al., 2023; Dai et al., 2023) and LLaVA (Liu H. et al., 2023; Liu et al., 2024)] together with text–voxel fusion modules inspired by Text-IF (Yi et al., 2024) and MultiAtt-RSSC (Cai et al., 2024). Following PaSCo (Cao A.-Q. et al., 2024) and HybridOcc (Zhao X. et al., 2024), we apply coarse-to-fine multi-level supervision in the decoder. Training losses are described in Section 3.7.

### 4.2 Evaluation results

We evaluate ESSC-RM as a refinement module on strong and weak SSC baselines and analyze its efficiency and qualitative behavior.

#### 4.2.1 Quantitative results

ESSC-RM is designed to prioritize voxel-wise semantic correctness (mIoU) over boundary-sensitive binary occupancy smoothness (IoU); therefore, minor IoU drops may accompany consistent mIoU gains.

##### 4.2.1.1 3D SSC performance

Table 1 reports SSC performance on SemanticKITTI, including representative image-based SSC baselines and our ESSC-RM variants. Among the listed baselines without ESSC-RM (upper block), DepthSSC (Yao et al., 2024) achieves the best SC-IoU (45.84%), while Symphonize (Jiang et al., 2024) attains the highest mIoU (14.89%). In addition to these method-level comparisons, we evaluate ESSC-RM as a plug-and-play refinement module on top of two representative SSC backbones: CGFormer (Tang et al., 2023) as a strong baseline (45.99% IoU, 16.87% mIoU) and MonoScene (Cao and de Charette, 2021) as a widely used weaker baseline (36.86% IoU, 11.08% mIoU). Due to training and storage overhead of voxel-level refinement, we instantiate ESSC-RM on these two backbones to demonstrate generality across different performance regimes; extending the plug-in evaluation to additional backbones is left for future work (see Section 5).

**Table 1:** Quantitative results on the SemanticKITTI validation set.

**Methods**	**IoU**	**mIoU**	** 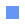 Car (3.92%)**	** 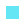 Bicycle (0.03%)**	** 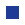 Motorcycle (0.03%)**	** 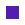 Truck (0.16%)**	** 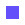 Other-vehicle (0.20%)**	** 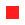 Person (0.07%)**	** 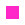 Bicyclist (0.07%)**	** 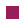 Motorcyclist (0.05%)**	** 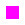 Road (15.30%)**	** 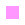 Parking (1.12%)**	** 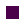 Sidewalk (11.13%)**	** 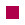 Other-ground (0.56%)**	** 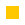 Building (14.10%)**	** 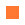 Fence (3.90%)**	** 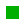 Vegetation (39.3%)**	** 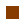 Trunk (0.51%)**	** 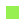 Terrain (9.17%)**	** 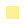 Pole (0.29%)**	** 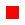 Traffic-sign (0.08%)**
**Baselines (without ESSC-RM)**																					
TPVFormer (Huang et al., 2023)	35.61	11.36	23.81	0.36	0.05	8.08	4.35	0.51	0.89	0.00	56.50	**20.60**	25.87	0.85	13.88	5.94	16.92	2.26	30.38	3.14	1.52
OccFormer (Zhang et al., 2023)	36.50	13.46	25.09	0.81	1.19	**25.53**	8.52	2.78	2.82	0.00	**58.85**	19.61	26.88	0.31	14.40	5.61	19.63	3.93	32.62	4.26	2.86
IAMSSC (Xiao et al., 2024b)	44.29	12.45	26.26	0.60	0.15	8.74	5.06	1.32	3.46	**0.01**	54.55	16.02	25.85	0.70	17.38	6.86	24.63	4.95	30.13	6.35	3.56
VoxFormer-S (Li Y. et al., 2023)	44.02	12.35	25.79	0.59	0.51	5.63	3.77	1.78	3.32	0.00	54.76	15.50	26.35	0.70	17.65	7.64	24.39	5.08	29.96	7.11	4.18
DepthSSC (Yao et al., 2024)	**45.84**	13.28	25.94	0.35	1.16	6.02	7.50	2.58	**6.32**	0.00	55.38	18.76	27.04	0.92	19.23	**8.46**	**26.37**	4.52	30.19	7.42	4.09
Symphonize (Jiang et al., 2024)	41.92	**14.89**	**28.68**	**2.54**	**2.82**	20.44	**13.89**	**3.52**	2.24	0.00	56.37	15.28	27.58	0.95	**21.64**	8.40	25.72	6.60	30.87	**9.57**	**5.76**
HASSC-S (Wang et al., 2024)	44.82	13.48	27.23	0.92	0.86	9.91	5.61	2.80	4.71	0.00	57.05	15.90	28.25	**1.04**	19.05	6.58	25.48	6.15	32.94	7.68	4.05
H2GFormer-S (Wang and Tong, 2024)	44.57	13.73	28.21	0.50	0.47	10.00	7.39	1.54	2.88	0.00	56.08	17.83	**29.12**	0.45	19.74	7.24	26.25	**6.80**	**34.42**	7.88	4.68
**MonoScene and ESSC-RM variants**																					
MonoScene (Cao and de Charette, 2021)	**36.86**	11.08	23.26	**0.61**	0.45	6.98	1.48	1.86	1.20	0.00	**56.52**	14.27	**26.72**	0.46	**14.09**	5.84	**17.89**	2.81	**29.64**	**4.14**	2.25
MonoScene + 3D U-Net	35.70	11.47	**23.46**	0.41	**0.87**	10.95	**3.69**	2.98	1.64	0.00	56.24	**14.95**	26.63	1.42	13.11	6.19	16.75	2.73	29.57	3.77	2.62
MonoScene + VLGM	35.62	11.49	22.76	0.44	0.71	**12.45**	3.12	**3.04**	1.64	0.00	56.48	14.35	26.64	1.42	13.55	**6.28**	16.44	**2.97**	29.50	3.85	2.65
MonoScene + PNAM	36.44	**11.51**	23.11	0.40	0.73	11.38	3.59	2.95	**1.69**	0.00	56.27	14.65	26.71	**1.45**	13.48	6.20	17.08	2.96	29.45	3.84	**2.69**
**CGFormer and ESSC-RM variants**																					
CGFormer (Tang et al., 2023)	**45.99**	16.87	34.32	4.61	2.71	19.44	7.67	2.38	4.08	0.00	65.51	20.82	**32.31**	**0.16**	**23.52**	**9.20**	**26.93**	8.83	**39.54**	10.67	**7.84**
CGFormer + 3D U-Net	43.53	17.17	33.99	5.28	**3.11**	22.39	8.22	2.65	4.05	0.00	65.29	20.26	32.14	0.13	23.11	8.93	26.84	11.17	38.99	**11.93**	**7.84**
CGFormer + VLGM	43.20	17.21	**34.33**	5.24	3.01	22.33	7.81	**2.70**	4.12	0.00	**65.52**	20.79	**32.31**	0.13	23.27	8.95	26.69	10.73	39.29	**11.93**	7.82
CGFormer + PNAM	44.33	**17.27**	34.11	**5.69**	2.94	**23.71**	**8.36**	2.64	**4.37**	0.00	65.27	**20.87**	31.90	**0.16**	22.70	9.08	26.63	**11.42**	38.91	11.78	7.66

The upper block lists baseline stereo-based SSC methods without ESSC-RM. The middle and lower blocks show MonoScene- and CGFormer-based ESSC-RM variants, respectively. Within each block, the best and second-best results are shown in bold and underlined, respectively.

To assess the generality of ESSC-RM, we plug it on top of both CGFormer and MonoScene, progressively adding (i) a plain 3D U-Net refinement head; (ii) the proposed neighborhood-attention-based refinement module (PNAM); and (iii) the vision–language guidance module (VLGM). The MonoScene and CGFormer blocks in Table 1 summarize these ablation results.

##### 4.2.1.2 ESSC-RM on CGFormer

As shown in the CGFormer block of Table 1, adding a 3D U-Net refinement head improves mIoU from 16.87 to 17.17%. Equipping the refinement with VLGM further increases mIoU to 17.21%, while PNAM achieves the best mIoU of 17.27% with only a modest IoU drop. The gains are more apparent on small and medium-scale categories (e.g., truck, bicycle, trunk, pole), suggesting that coarse-to-fine decoding and neighborhood-aware aggregation help correct local ambiguities and recover thin structures that are challenging for the backbone alone.

Despite consistent improvements, the absolute mIoU gain on CGFormer remains moderate (from 16.87 to 17.27%, +0.40, i.e., ~2% relative). This is mainly because ESSC-RM performs refinement *in the voxel-prediction space* by design: it takes the discrete semantic occupancy $\hat{\text{Y}}$ predicted by the backbone, embeds it into a continuous feature map, and refines it via a 3D U-Net style encoder–decoder (with PNAM/VLGM as optional enhancements). Consequently, the global occupancy layout and object extents remain largely inherited from the backbone prediction, while ESSC-RM mainly improves local semantic consistency and boundary delineation (e.g., thin objects and class-confusing regions), which naturally limits the headroom when the backbone output is already geometrically plausible.

##### 4.2.1.3 ESSC-RM on MonoScene

The MonoScene block of Table 1 shows that ESSC-RM also improves the weaker MonoScene baseline. VLGM increases mIoU from 11.08 to 11.49%, and PNAM further pushes it to 11.51% with comparable IoU. These consistent gains across CGFormer and MonoScene support the plug-and-play nature of ESSC-RM, indicating that the refinement is not tied to a specific SSC backbone.

On MonoScene, ESSC-RM improves mIoU from 11.08 to 11.51% (+0.43, ~4% relative). Since the refinement module does not introduce additional sensor-level geometric observations beyond the backbone output, its improvement is mainly achieved by enforcing multi-scale voxel consistency and reducing local misclassifications. When large missing structures are completely absent in $\hat{\text{Y}}$, *post-hoc* voxel refinement cannot fully recover them, whereas it remains effective at sharpening boundaries and improving local semantic coherence.

##### 4.2.1.4 SC-IoU trade-off

Although refinement improves mIoU, SC-IoU (occupied vs. free) can slightly decrease in some cases; this is an expected design trade-off rather than a flaw. For example, on CGFormer, ESSC-RM increases mIoU by +0.40 (16.87% → 17.27%) while SC-IoU decreases by 1.66 (45.99% → 44.33%). This behavior is expected because SC-IoU is a *binary* occupancy metric that is particularly sensitive to boundary voxels: semantic refinement around thin structures and object borders may flip a small fraction of occupied/free decisions, increasing FP/FN near boundaries even when per-class semantics improve. As SC-IoU aggregates over the entire occupancy grid, such boundary perturbations can lead to a measurable IoU change, reflecting a mild trade-off between semantic correction (mIoU) and boundary-sensitive binary occupancy under discrete voxel predictions.

##### 4.2.1.5 Refinement module efficiency

We further analyze the computational overhead of ESSC-RM on top of CGFormer (Table 2). CGFormer itself has 122.42 M parameters, requires about 19.3 GB memory during training and 6.55 GB at inference, and runs at approximately 205 ms per frame. The 3D U-Net refinement head adds only 13.36 M parameters and can be trained jointly with CGFormer on a 24 GB GPU when the backbone is set to inference mode. VLGM and PNAM increase parameter counts and inference time more noticeably, but remain practical for offline refinement or two-stage pipelines.

**Table 2:** Ablation study on the efficiency of the refinement module with CGFormer as backbone.

**Model**	**IoU**	**mIoU**	**Params (M)**	**Train memory (M)**	**Infer. memory (M)**	**Infer. time (ms)**
CGFormer	**45.99**	16.87	122.42	19,330	6,550	205
+3D U-Net	43.53	17.17	13.36	12,726	4,904	215
+VLGM	43.20	17.21	43.96	18,942	5,382	340
+PNAM	44.33	**17.27**	9.59	20,664	5,042	265

Bold values indicate the best performance (highest is best) within each comparison group.

### 4.2.2 Qualitative results

**Figure 7:**
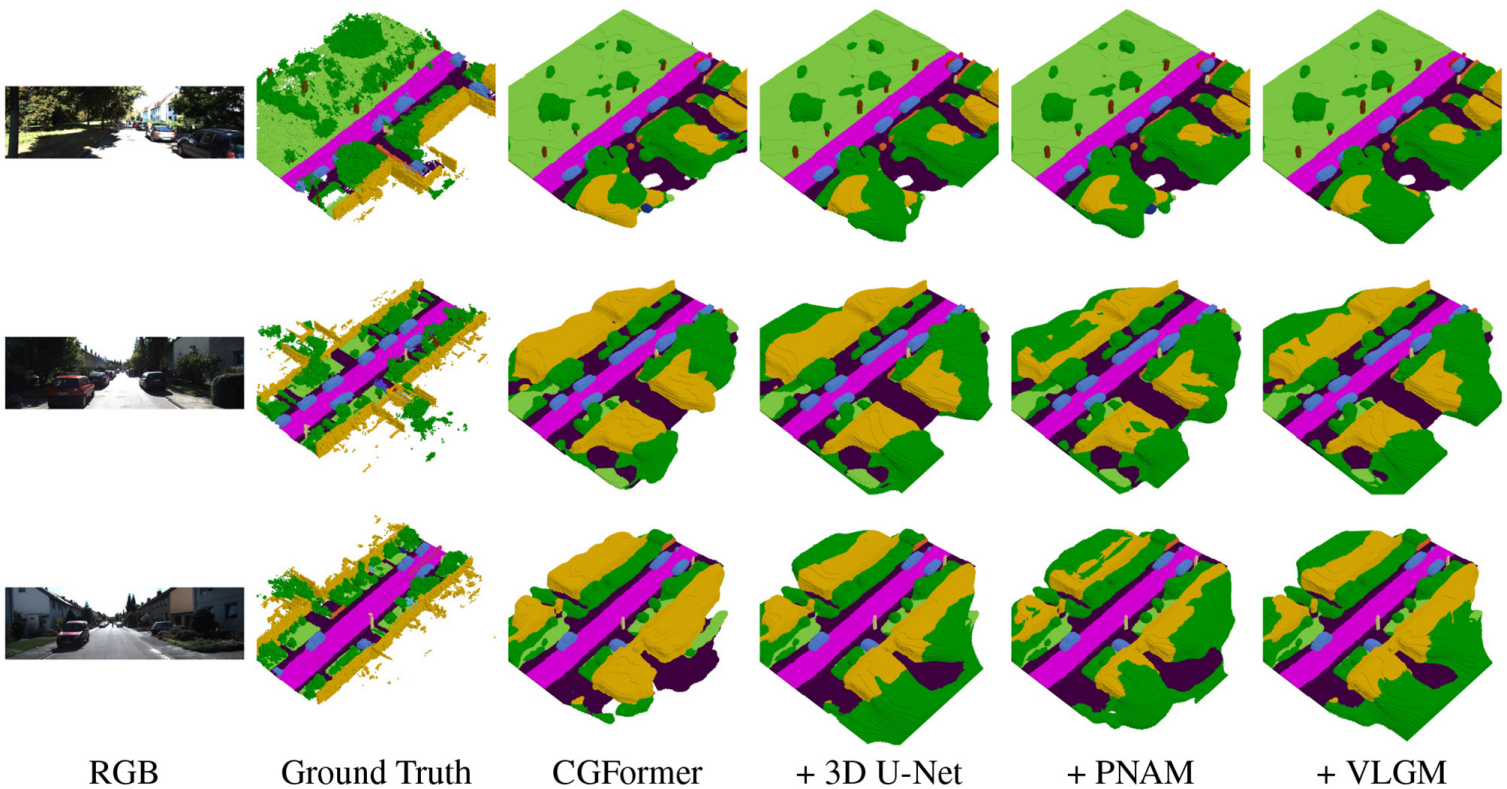
Qualitative results of ESSC-RM on CGFormer (Tang et al., 2023) on the SemanticKITTI (Behley et al., 2019, 2021) validation set. ESSC-RM progressively refines the baseline prediction, filling missing regions and improving object shapes and small semantic structures.

**Figure 8:**
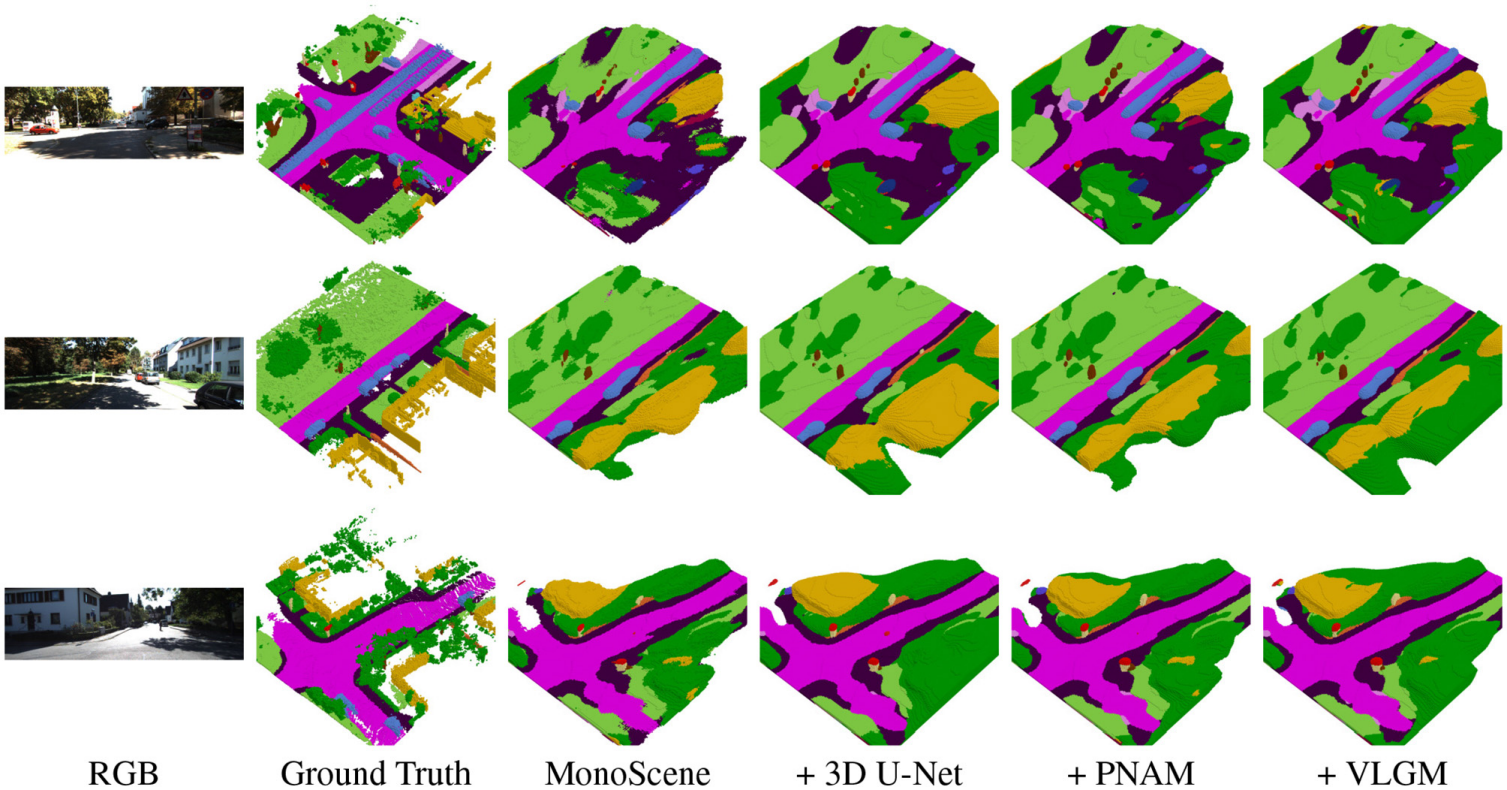
Qualitative results of ESSC-RM on MonoScene (Cao and de Charette, 2021) on the SemanticKITTI (Behley et al., 2019, 2021) validation set. ESSC-RM enhances the baseline prediction by completing missing structures, improving geometric consistency, and sharpening small semantic objects.

Figures 7, 8 present qualitative results of ESSC-RM applied respectively to CGFormer and MonoScene on the SemanticKITTI validation set. Each row displays the input RGB image, ground truth, the prediction of the baseline model, and the refined outputs after integrating the 3D U-Net, PNAM, and VLGM modules.

Across both baselines, the refinement module consistently reduces holes and misclassifications in occluded or boundary regions, restores missing vegetation and structures at scene edges, and produces smoother and more coherent semantic layouts. On large-scale structures such as roads and buildings, PNAM and VLGM further improve geometric regularity, yielding cleaner contours and more stable surface predictions. For small-scale objects like traffic signs and poles, text-derived priors in VLGM highlight distinctive semantic regions, while PNAM enhances local aggregation and sharpens object boundaries.

These results demonstrate that ESSC-RM provides robust and generalizable refinement across different SSC backbones.

## 5 Conclusion

In summary, while ESSC-RM improves semantic scene completion by refining voxel predictions with PNAM and VLGM, several challenges remain. First, the refinement module still relies on 3D convolutions and attention, leading to non-negligible latency and memory overhead. Second, our evaluation is currently centered on SemanticKITTI, and broader generalization remains constrained by differences in voxel resolution, label taxonomy, and scene layout, which may require re-training or lightweight structural adaptation. Moreover, while we validate the plug-and-play behavior on two representative SSC backbones (CGFormer and MonoScene), extending the plug-in evaluation to additional backbones is still limited by the training and storage overhead of voxel-level refinement. Third, PNAM and VLGM are incorporated as largely independent components without a unified fusion mechanism, and emphasizing semantic correction may slightly compromise geometric completeness, resulting in minor degradations in SC-IoU.

Future work will therefore explore lightweight and efficient representations (e.g., sparse convolution, tri-plane features, and Gaussian voxelization), knowledge distillation for compact deployment, as well as structured pruning and quantization-aware optimization to further reduce latency and memory footprint. We will also investigate adapter-based transfer across datasets, and broaden plug-in evaluation across diverse SSC backbones to further substantiate generality. In addition, we will study adaptive fusion layers that more tightly couple local geometric attention with textual priors. Finally, integrating generative priors (e.g., CVAE- or diffusion-based models) to pre-complete sparse voxels, together with extensive evaluation on diverse real-world benchmarks, may further improve the robustness, practicality, and scalability of ESSC-RM.

## Data Availability

Publicly available datasets were analyzed in this study. This data can be found at: https://semantic-kitti.org/.
