# Correction: Enhancing 3D semantic scene completion with refinement module

**DOI:** 10.3389/fnbot.2026.1838975

**Published:** 2026-04-07

**Authors:** Frontiers Production Office

**Affiliations:** Frontiers Media SA, Lausanne, Switzerland

**Keywords:** plug-and-play, PNAM, refinement, semantic scene completion, vison-language guidance

An incorrect DOI (10.3389/fnbot.2025.1768219) was registered for this article on publication. The correct DOI (10.3389/fnbot.2026.1768219) has now been registered.

An incorrect publication date collection year of “2025” was also assigned in the XML instead of the correct publication date collection year “2026”.

The original version of this article has been updated.

