# Supplementary material for: Enhancing 3D semantic scene completion with refinement module
Source: Front Neurorobot. 2026 Mar 6;20:1768219. doi: 10.3389/fnbot.2026.1768219 (PMC13082174; doi:10.3389/fnbot.2026.1768219)
Supplement: Supplementary file 1 [file Data_Sheet_1.pdf]

Supplementary Material

1 ABLATION STUDY

This appendix reports additional ablation studies conducted to understand the effect of individual components in ESSC-RM. We investigate (i) feature enhancement block (FEB) design, (ii) multi-scale supervision, (iii) choices of vision–language models (VLMs), text semantic encoders, and fusion strategies, and (iv) the influence of learning rate and loss combinations. All experiments use CGFormer Yu et al. (2024) as the base semantic scene completion model unless otherwise specified.

1.1 Feature Enhancement Block (FEB)

ESSC-RM employs a 3D U-Net encoder with FEBs following SemCity Lee et al. (2024). Each FEB consists of an initial ConvBlock, a spatial downsampling layer, and an optional post-downsampling ConvBlock. To analyze how these architectural choices affect feature quality, two dimensions are ablated: (1) MaxPooling vs. convolutional downsampling, and (2) whether a second ConvBlock is appended.

Table S1 shows that convolutional downsampling provides slightly higher mIoU than MaxPooling, and adding a second ConvBlock further improves performance for both 3D U-Net and PNAM variants. These gains come at a small cost in IoU and computational overhead, indicating a trade-off between representational richness and efficiency.

Model	FEB Components				IoU	mIoU
	ConvBlock1	MaxPooling	ConvDown	ConvBlock2		
CGFormer + 3D U-Net	✓	✓			44.15	17.03
CGFormer + 3D U-Net	✓		✓		43.48	17.06
CGFormer + 3D U-Net	✓	✓		✓	43.43	17.13
CGFormer + 3D U-Net	✓		✓	✓	43.53	**17.17**
CGFormer + PNAM	✓	✓			44.85	17.19
CGFormer + PNAM	✓		✓		44.05	17.09
CGFormer + PNAM	✓	✓		✓	44.88	17.26
CGFormer + PNAM	✓		✓	✓	44.33	**17.27**

Table S1. Ablation study on the Feature Enhancement Block (FEB) following SemCity Lee et al. (2024).

1.2 Multi-scale Supervision

We evaluate whether supervising voxel predictions at multiple resolutions improves semantic consistency. Following the multi-scale design in PaSCo Cao et al. (2024), we compare training with only $1:1$ supervision against multi-scale supervision at $\{1:8, 1:4, 1:2, 1:1\}$.

Across all refinement modules, multi-scale supervision improves mIoU (e.g., 16.87%→17.21% for VLGM and 17.07%→17.27% for PNAM), while IoU decreases slightly. This suggests that supervising intermediate resolutions strengthens fine-grained semantic discrimination.

Model	Multi-scale Supervision 1:8	1:4	1:2	1:1	IoU	mIoU
CGFormer + 3D U-Net				✓	44.21	16.98
CGFormer + 3D U-Net	✓	✓	✓	✓	43.53	**17.17**
CGFormer + VLGM				✓	44.09	16.87
CGFormer + VLGM	✓	✓	✓	✓	43.20	**17.21**
CGFormer + PNAM				✓	44.44	17.07
CGFormer + PNAM	✓	✓	✓	✓	44.33	**17.27**

Table S2. Ablation study on multi-scale supervision following PaSCo Cao et al. (2024).

1.3 Text Annotation Generation

Two VLMs are compared for generating scene descriptions: InstructBLIP Dai et al. (2023) and LLaVA Liu et al. (2023, 2024). As shown in Figure S1 and Table S3, LLaVA generates considerably longer descriptions (average 182 words vs. 78), often with narrative-style content, whereas InstructBLIP produces concise object-focused descriptions with more accurate attributes.

InstructBLIP is based on BLIP-2 Li et al. (2023) with Vicuna-7B as the LLM, while LLaVA uses a ViT-based visual encoder and a larger Yi-34B LLM. The larger language model in LLaVA results in much higher inference cost (42.25s vs. 13.67s per frame). Therefore, InstructBLIP is preferable for on-the-fly annotation, while LLaVA is mainly used offline to evaluate the effect of richer textual priors.

VLM	Vision Encoder	LLM	Inference Time
InstructBLIP	ViT-G/14	Vicuna-7B	13.67s
LLaVA	ViT-L/14	Yi-34B	42.25s

Table S3. Comparison of VLM architectures and inference latency for InstructBLIP Dai et al. (2023) and LLaVA Liu et al. (2023, 2024).

1.4 Text Semantic Encoder

We evaluate several text encoders using texts from both VLMs: Q-Former from BLIP-2 Li et al. (2023), CLIP Radford et al. (2021), LongCLIP Zhang et al. (2024), and JinaCLIP/JinaCLIP_1024 Xiao et al. (2024); Koukounas et al. (2024). Table S4 shows that Q-Former combined with DCAM yields the highest mIoU for both InstructBLIP (17.18%) and LLaVA (17.21%). Higher-dimensional encoders do not consistently improve mIoU, suggesting that fusion quality matters more than feature dimensionality.

1.5 Text Semantic Fusion Strategy

We examine where to inject text semantics within the refinement module. Figure S2 illustrates representative fusion schemes following BiPVL-style designs Sultan et al. (2025). Since the text encoder is frozen, bidirectional encoder fusion is not applicable and late fusion conflicts with skip connections. We therefore test encoder-only, decoder-only, and encoder+decoder fusion.

Encoder-only fusion gives the highest IoU, whereas decoder-only fusion slightly improves mIoU. The best mIoU is obtained when fusing at both encoder and decoder, indicating complementary benefits from low-level and high-level semantic guidance (Table S5).

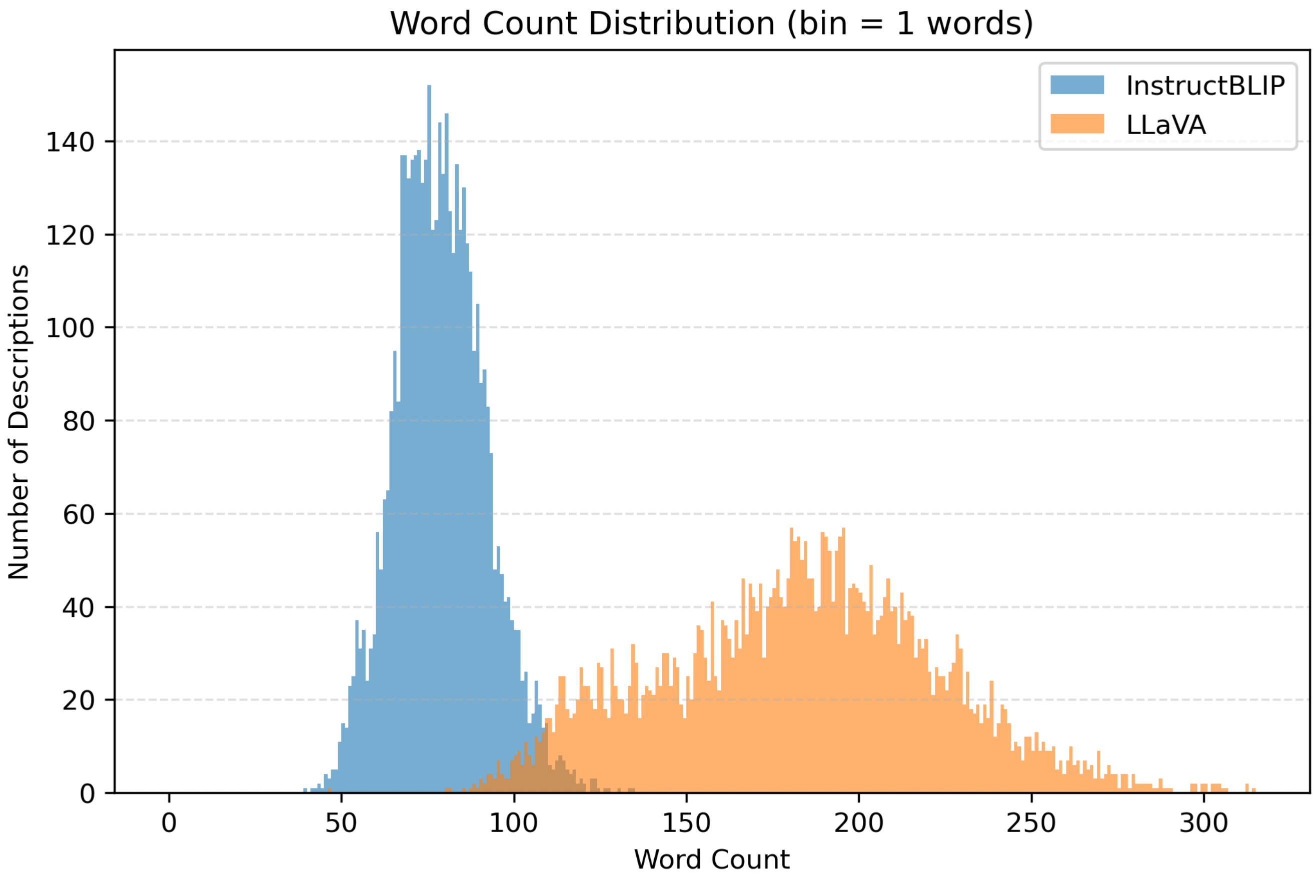

Figure S1: Word count distribution of text outputs from InstructBLIP Dai et al. (2023) and LLaVA Liu et al. (2023, 2024).

Baseline	VLM	Text Encoder	Feature Dim.	Fuser	IoU	mIoU
CGFormer	InstructBLIP	Q-Former Li et al. (2023)	$N \times L \times 256$	DCAM	44.02	**17.18**
		CLIP Radford et al. (2021)	$N \times 512$	SIGM	44.10	17.11
		LongCLIP Zhang et al. (2024)	$N \times 768$	SIGM	44.18	17.04
		JinaCLIP Xiao et al. (2024); Koukounas et al. (2024)	$N \times 512$	SIGM	42.83	17.11
		JinaCLIP_1024 Xiao et al. (2024); Koukounas et al. (2024)	$N \times 1024$	SIGM	43.26	17.07
CGFormer	LLaVA Liu et al. (2023, 2024)	Q-Former Li et al. (2023)	$N \times L \times 256$	DCAM	43.20	**17.21**
		CLIP Radford et al. (2021)	–	–	–	–
		LongCLIP Zhang et al. (2024)	–	–	–	–
		JinaCLIP Xiao et al. (2024); Koukounas et al. (2024)	$N \times 512$	SIGM	43.51	17.11
		JinaCLIP_1024 Xiao et al. (2024); Koukounas et al. (2024)	$N \times 1024$	SIGM	43.88	17.08

Table S4. Ablation study on text semantic encoders, including Q-Former Li et al. (2023), CLIP Radford et al. (2021), LongCLIP Zhang et al. (2024), and JinaCLIP variants Xiao et al. (2024); Koukounas et al. (2024).

1.6 Learning Rate

We evaluate three learning rates (3×10^{-4}, 1×10^{-4}, 5×10^{-5}). As shown in Table S6, all refinement modules achieve their best mIoU with the smallest learning rate. Since the refinement module updates an already well-formed 3D representation from CGFormer Yu et al. (2024), a smaller step size helps stabilize optimization and prevents overfitting.

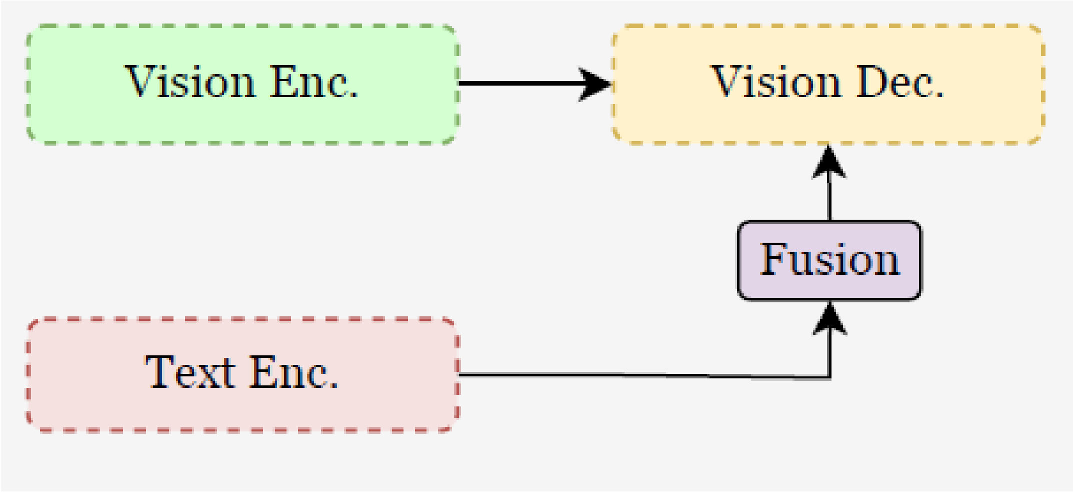

Figure S2: Illustration of text–vision fusion strategies inspired by BiPVL Sultan et al. (2025).

Baseline	VLM	Encoder Fusion	Decoder Fusion	IoU	mIoU
CGFormer	InstructBLIP Dai et al. (2023)	✓		44.40	17.10
			✓	44.23	17.13
		✓	✓	44.02	**17.18**
CGFormer	LLaVA Liu et al. (2023, 2024)	✓		43.87	17.08
			✓	43.55	17.16
		✓	✓	43.20	**17.21**

Table S5. Ablation study on text semantic fusion placement, comparing encoder-only, decoder-only, and encoder+decoder fusion strategies Sultan et al. (2025).

Model	3e-4	1e-4	5e-5	IoU	mIoU
CGFormer + 3D U-Net	✓			45.71	16.87
CGFormer + 3D U-Net		✓		44.72	16.92
CGFormer + 3D U-Net			✓	43.53	**17.17**
CGFormer + VLGM	✓			45.46	17.05
CGFormer + VLGM		✓		44.52	17.03
CGFormer + VLGM			✓	43.20	**17.21**
CGFormer + PNAM	✓			45.18	17.13
CGFormer + PNAM		✓		44.75	17.16
CGFormer + PNAM			✓	44.33	**17.27**

Table S6. Ablation study on learning rate for ESSC-RM built on CGFormer Yu et al. (2024).

1.7 Loss Design

Finally, we test the impact of adding Lovász-softmax loss Berman and Blaschko (2017) on top of the default cross-entropy (CE) and SCAL. Lovász-softmax has been adopted in several recent SSC works Wang et al. (2026); Liang et al. (2025); Kim et al. (2025) due to its direct optimization of an IoU surrogate. Table S7 shows that introducing Lovász-softmax slightly decreases mIoU for all refinement modules and does not improve IoU. SCAL provides strong voxel-level consistency supervision, and additional IoU-oriented losses do not yield further gains on SemanticKITTI Behley et al. (2019, 2021).

Model	CE	SCAL	Lovász-softmax Berman and Blaschko (2017)	IoU	mIoU
CGFormer + 3D U-Net	✓	✓		43.53	**17.17**
CGFormer + 3D U-Net	✓	✓	✓	44.15	16.94
CGFormer + VLGM	✓	✓		43.20	**17.21**
CGFormer + VLGM	✓	✓	✓	42.84	17.12
CGFormer + PNAM	✓	✓		44.33	**17.27**
CGFormer + PNAM	✓	✓	✓	43.74	17.18

Table S7. Ablation study on loss configurations, including Lovász-softmax Berman and Blaschko (2017) as in Wang et al. (2026); Liang et al. (2025); Kim et al. (2025).

REFERENCES

Behley, J., Garbade, M., Milioto, A., Quenzel, J., Behnke, S., Gall, J., et al. (2021). Towards 3d lidar-based semantic scene understanding of 3d point cloud sequences: The semantickitti dataset. *The International Journal of Robotics Research* 40, 959–967. doi:10.1177/02783649211006735

Behley, J., Garbade, M., Milioto, A., Quenzel, J., Behnke, S., Stachniss, C., et al. (2019). A dataset for semantic segmentation of point cloud sequences. *CoRR* abs/1904.01416

Berman, M. and Blaschko, M. B. (2017). Optimization of the jaccard index for image segmentation with the lovász hinge. *CoRR* abs/1705.08790

Cao, A.-Q., Dai, A., and de Charette, R. (2024). Pasco: Urban 3d panoptic scene completion with uncertainty awareness. In *CVPR*

Dai, W., Li, J., LI, D., Tiong, A., Zhao, J., Wang, W., et al. (2023). Instructblip: Towards general-purpose vision-language models with instruction tuning. In *Advances in Neural Information Processing Systems*, eds. A. Oh, T. Naumann, A. Globerson, K. Saenko, M. Hardt, and S. Levine (Curran Associates, Inc.), vol. 36, 49250–49267

Kim, J., Kang, C., Lee, D., Choi, S., and Choi, J. W. (2025). Protoocc: Accurate, efficient 3d occupancy prediction using dual branch encoder-prototype query decoder. In *Proceedings of the AAAI Conference on Artificial Intelligence*. vol. 39, 4284–4292

[Dataset] Koukounas, A., Mastrapas, G., Wang, B., Akram, M. K., Eslami, S., Günther, M., et al. (2024). jina-clip-v2: Multilingual multimodal embeddings for text and images

Lee, J., Lee, S., Jo, C., Im, W., Seon, J., and Yoon, S.-E. (2024). Semcity: Semantic scene generation with triplane diffusion. In *Proceedings of the IEEE/CVF conference on computer vision and pattern recognition*. doi:10.48550/arXiv.2403.07773

Li, J., Li, D., Savarese, S., and Hoi, S. (2023). Blip-2: bootstrapping language-image pre-training with frozen image encoders and large language models. In *Proceedings of the 40th International Conference on Machine Learning* (JMLR.org), ICML'23

Liang, L., Akhtar, N., Vice, J., Kong, X., and Mian, A. S. (2025). Skip mamba diffusion for monocular 3d semantic scene completion. *Proceedings of the AAAI Conference on Artificial Intelligence* 39, 5155–5163. doi:10.1609/aaai.v39i5.32547

Liu, H., Li, C., Li, Y., and Lee, Y. J. (2024). Improved baselines with visual instruction tuning. In *2024 IEEE/CVF Conference on Computer Vision and Pattern Recognition (CVPR)*. 26286–26296. doi:10.1109/CVPR52733.2024.02484

Liu, H., Li, C., Wu, Q., and Lee, Y. J. (2023). Visual instruction tuning. In *Proceedings of the 37th International Conference on Neural Information Processing Systems* (Red Hook, NY, USA: Curran Associates Inc.), NIPS '23

Radford, A., Kim, J. W., Hallacy, C., Ramesh, A., Goh, G., Agarwal, S., et al. (2021). Learning transferable visual models from natural language supervision. In *Proceedings of the 38th International Conference on Machine Learning*, eds. M. Meila and T. Zhang (PMLR), vol. 139 of *Proceedings of Machine Learning Research*, 8748–8763

Sultan, R. I., Zhu, H., Li, C., and Zhu, D. (2025). Bipvl-seg: Bidirectional progressive vision-language fusion with global-local alignment for medical image segmentation. *arXiv preprint arXiv:2503.23534*

Wang, M., Wu, F., Qin, Y., Li, R., Tang, Z., and Li, K. (2026). Vision-based 3d semantic scene completion via capture dynamic representations. *Knowledge-Based Systems* 331, 114550. doi:https://doi.org/10.1016/j.knosys.2025.114550

Xiao, H., Mastrapas, G., and Wang, B. (2024). Jina CLIP: Your CLIP model is also your text retriever. In *Multi-modal Foundation Model meets Embodied AI Workshop @ ICML2024*

Yu, Z., Zhang, R., Ying, J., Yu, J., Hu, X., Luo, L., et al. (2024). Context and geometry aware voxel transformer for semantic scene completion. In *Advances in Neural Information Processing Systems*. vol. 37, 1531–1555

Zhang, B., Zhang, P., Dong, X., Zang, Y., and Wang, J. (2024). Long-clip: Unlocking the long-text capability of clip. In *Computer Vision – ECCV 2024*, eds. A. Leonardis, E. Ricci, S. Roth, O. Russakovsky, T. Sattler, and G. Varol (Cham: Springer), vol. 15109 of *Lecture Notes in Computer Science*. doi:10.1007/978-3-031-72983-6_18
